# High Performance Solution Processed Organic Field Effect Transistors with Novel Diketopyrrolopyrrole-Containing Small Molecules

**DOI:** 10.1038/s41598-017-00277-7

**Published:** 2017-03-13

**Authors:** Bogyu Lim, Huabin Sun, Jaechol Lee, Yong-Young Noh

**Affiliations:** 1 Future Technology Research Center, Corporate R&D, LG Chem Research Park, 188, Moonji-ro, Yuseong-gu, Daejeon 34122 Republic of Korea; 2 Department of Energy and Materials Engineering, Dongguk University, 30 Pildong-ro, 1-gil, Jung-gu, Seoul, 04620 Republic of Korea

## Abstract

The donor-acceptor (D-A)-type diketopyrrolopyrrole (DPP)-based small molecules (LGC-D117 and LGC-D118) were synthesized and used as the active layer of solution-processable organic field-effect transistors (OFETs). Both LGC-D117 and LGC-D118 contain silaindacenodithiophene as electron-donor units with DPP as an electron-accepting linker, and octylrhodanine as the electron-accepting end group. The molecules were functionalized with different side chains to study their effects on OFET characteristics. LGC-D117 has a simple branched alkyl side chain, whereas LGC-D118 features a bulky siloxane-terminated hybrid alkyl chain. The siloxane side chains of LGC-D118 account for its better crystallinity, leading to significantly high field-effect mobility (max 3.04 cm^2^ V^−1^ s^−1^). In particular, LGC-D118 is well soluble and sustains the high mobility in the environmentally friendly 2-methyltetrahydrofuran solvent with low temperature annealing at 100 °C due to the bulky siloxane-terminated alkyl side chain.

## Introduction

Conjugated molecules have been actively researched due to their great potential for realizing ultra-lightweight flexible devices on plastic substrates^1–5^. In particular, side chain engineering of these molecules enables device fabrication using cost-effective graphic-art printing processes^6–12^. Among the emerging organic devices, organic field-effect transistors (OFETs) are considered a core component of various analog and digital integrated circuits. The realization of high-performance OFETs requires the development of novel organic semiconductors (OSCs) with excellent π-orbital planarity and small intermolecular distance to facilitate inter- and intramolecular charge carrier transport in the transistor channel^13–18^. Conjugated polymers composed of alternating electron donor (D) and acceptor (A) moieties (D-A) show impressively high field-effect mobilities (*μ*_FET_)^19–23^. Strong attractive interactions between the D units in one molecule and the A units in the neighboring one(s) provide a large π-orbital transfer integral for efficient charge carrier hopping^24^. Recently D-A conjugated polymers have shown rapid progress of their charge carrier mobility, showing values above 10 cm^2^ V^−1^ s^−1^ for both holes and electrons due to the active utilization of various large planar building blocks, such as isoindigo, naphthalenediimide, perylenediimide, and diketopyrrolopyrrole (DPP)^8, 19, 25–29^.

Despite the impressive success of D-A polymers, the development of D-A small molecules for the active layer of solution-processable OFETs lags behind. Solution-processable small-molecule OSCs have several advantages over polymer semiconductors, exhibiting less batch-to-batch variation (i.e., better reproducibility), easy purification, and functionalization potential. Thus, the active development of solution-processable D-A small molecules is essential^20^. In particular, DPP-based D-A small molecules have been widely investigated because of their promising performance in organic electronic applications^30, 31^. Zhou *et al*.^32^ proposed a new D-A small-molecule OSC that features a tetrathienoacene donor core with DPP as the end acceptor and exhibits hole mobility in the range of 0.02–0.09 cm^2^ V^−1^ s^−1^. Kim *et al*.^33, 34^ also used DPP as an acceptor and dithiensilole as the core donor, achieving ambipolar transport characteristics with hole mobility of 0.011 cm^2^ V^−1^ s^−1^ and electron mobility of 0.015 cm^2^ V^−1^ s^−1^. However, OFETs utilizing DPP-based small molecules show a much lower performance than those utilizing DPP-based polymers.

In addition to the main backbone design, side chain engineering is also important for OFET performance, determining solubility, molecular packing, polarity, and film forming properties. In particular, molecular packing is greatly affected by alkyl chain length and branching point position^35^. Nevertheless, a limited number of side chain designs have been reported, featuring mostly linear and branched hydrocarbon alkyl chains of different lengths^10, 36–38^. For example, Minari *et al*.^39^ studied the Alkyl chain length dependence of OFET performance. Compared to branched alkyl chains, linear alkyl chains generally promote closer molecular packing due to interchain interdigitation^40, 41^. However, DPP based semiconductors utilizing linear alkyl chains frequently show poor or no solubility in common solvents. Recently, Mei *et al*.^42^ demonstrated OFETs with improved performance employing bulky siloxane-terminated linear alkyl chains in the polymer, the enhancement can be ascribed to combination of smaller π-stacking distances and larger crystalline coherence length. Lee *et al*. also reported highly improved ambipolar OFET performance for DPP based polymer with bulky siloxane-terminated linear alkyl chains^43^.

**Figure 1:**
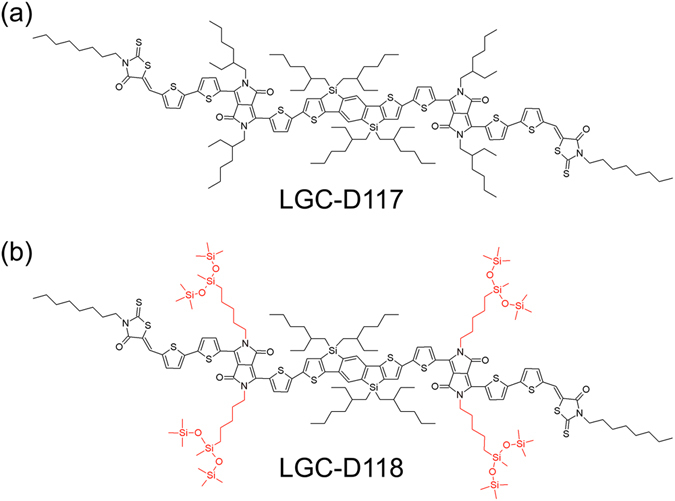
Molecular structures of LGC-D117 and LGC-D118.

In this study, we report and compare two new D-A type DPP-based small molecules, LGC-D117 and LGC-D118 (Fig. 1) for the active layer of solution-processable OFETs. LGC-D117 and LGC-D118 feature silaindacenodithiophene as the electron-donating core, DPP as the electron-accepting linker, and octylrhodanine as the electron-accepting end group. Both LGC-D117 and LGC-D118 have the same backbone, but the side chains attached to the DPP linker are different. Because of the change from a normal alkyl chain to a bulky siloxane-terminated hybrid alkyl chain^42, 43^, LGC-D118 exhibited improved crystallinity even in case of the as-spun film, leading to a significantly enhanced *μ*_FET_ after annealing at 100 °C (up to 3.04 cm^2^ V^−1^ s^−1^). To the best of our knowledge, this is the first report on siloxane-terminated hybrid bulky side chain in small molecular OSCs for OFETs. Interestingly, this side chain makes the semiconductor film highly crystalline, accounting for its rather high mobility (max 3.04 cm^2^ V^−1^ s^−1^) with low temperature annealing at 100 °C, which is the highest field-effect hole mobility reported in literature for DPP-based solution-processable small-molecule OSCs. Additionally, the bulky side chain makes LGC-D118 readily soluble in various eco-friendly solvents such as 2-methyltetrahydrofuran (M-THF). The LGC-D118-based OFETs fabricated using the M-THF solution showed impressive (max 2.60 cm^2^ V^−1^ s^−1^) after low temperature annealing at 100 °C. These results indicate that the newly synthesized small molecule OSC with siloxane-terminated hybrid bulky side chain, LGC-D118, can be a promising active material for high-performance flexible OFETs with low temperature processes for the commercialization.

## Results

LGC-D117 and LGC-D118 were obtained via palladium-catalyzed coupling and Knoevenagel condensation, and were characterized by ^1^H NMR and mass spectroscopy, with synthetic routes and detailed procedures provided in the Supporting Information. The new OSCs exhibit good solubility in common organic solvents such as chloroform, chlorobenzene, and 1,2-dichlorobenzene, with LGC-D118 displaying better solubility than LGC-D117 due to the siloxane-terminated hybrid bulky side chain. Differential scanning calorimetry (DSC) analysis of LGC-D117 and LGC-D118 revealed sharp endotherms at 218 and 248 °C, while exotherms were detected at 168 and 231 °C, respectively, as shown in Fig. S1 and summarized in Table 1. Due to stiff siloxane side chain, LGC-D118 has higher melting and crystallized temperature compared with LGC-D117.

**Table 1:** Thermal, optical, and electrochemical properties of LGC-D117 and LGC-D118.

Material	*T*_m_^a^ (°C)	*T*_c_^b^ (°C)	*λ*_max,sol_^c^ (nm)	*λ*_max,film,as_^d^ (nm)	*λ*_max,film,ann_^e^ (nm)	HOMO^f^ (eV)	LUMO (eV)	*E*_g,UV_^g^ (eV)	*E*_g,CV_^h^ (eV)
LGC-D117	218	168	710	765	778	−5.19	−3.60	1.41	1.59
LGC-D118	248	231	710	772	770	−5.18	−3.66	1.40	1.52

^a^Melting temperature. ^b^Crystallization temperature. ^c^Measurements in chlorobenzene solution. ^d^Measurements in films were spin-casted on the glass before annealing. ^e^Measurements in films were spin-casted on the glass after annealing. ^f^The HOMO level was estimated from cyclic voltammetry measurement. ^g^Optical band gap was determined from onset of the absorption in film. ^h^Electrochemical band gap was estimated by cyclic voltammetry measurement.

**Figure 2:**
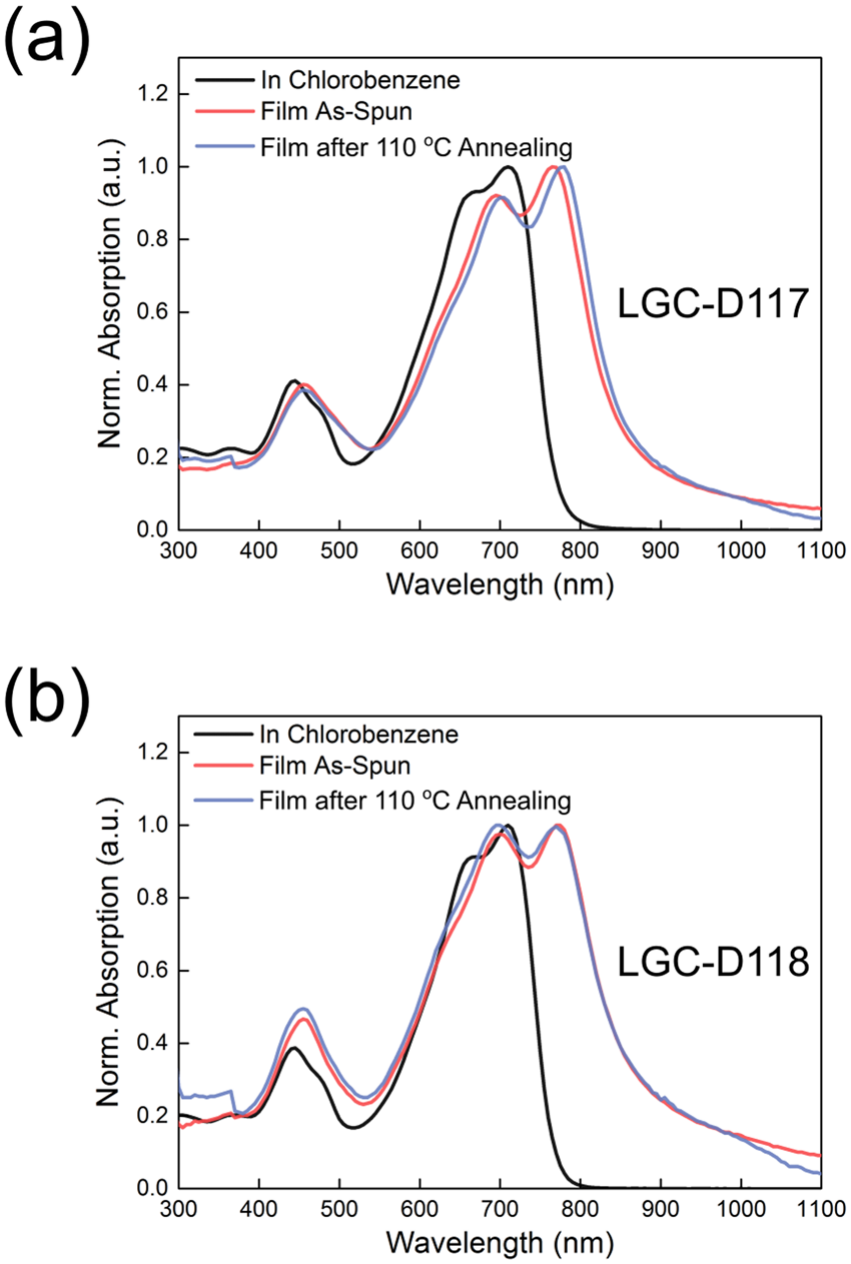
UV-vis absorption spectra of (**a**) LGC-D117, (**b**) LGC-D118 solutions and films.

The UV-vis spectra of LGC-D117 and LGC-D118 as chlorobenzene solutions and thin films are shown in Fig. 2, with the corresponding absorption properties summarized in Table 1. The absorption spectra of all compounds were similar for both solutions and films. The optical bandgaps of LGC-D117, and LGC-D118 were estimated as 1.41 and 1.40 eV, respectively. Both LGC-D117 and LGC-D118 exhibited similar absorption peaks around 710 nm in solution, whereas the absorption peak of the LGC-D118 film was more red-shifted than that of the LGC-D117 film, indicating stronger π–π stacking in the LGC-D118 film. After annealing at 110 °C, however, the absorption peak of the LGC-D117 film was slightly red-shifted, while that of LGC-D118 was marginally blue-shifted. The electrochemical properties of all compounds were investigated by cyclic voltammetry, as shown in Fig. S2 and summarized in Table 1. The lowest unoccupied molecular orbital (LUMO) and the highest occupied molecular orbital (HOMO) energy levels were calculated using the onset of the reduction/oxidation peaks. The LUMO energy levels of LGC-D117 and LGC-D118 were estimated as −3.60 and −3.66 eV, respectively, based on the onset reduction potential referenced to ferrocenium/ferrocene (−4.8 eV). The respective HOMO energy levels were estimated as −5.19, and −5.18 eV, while the electrochemical bandgaps were calculated as 1.59 and 1.52 eV, respectively.

**Figure 3:**
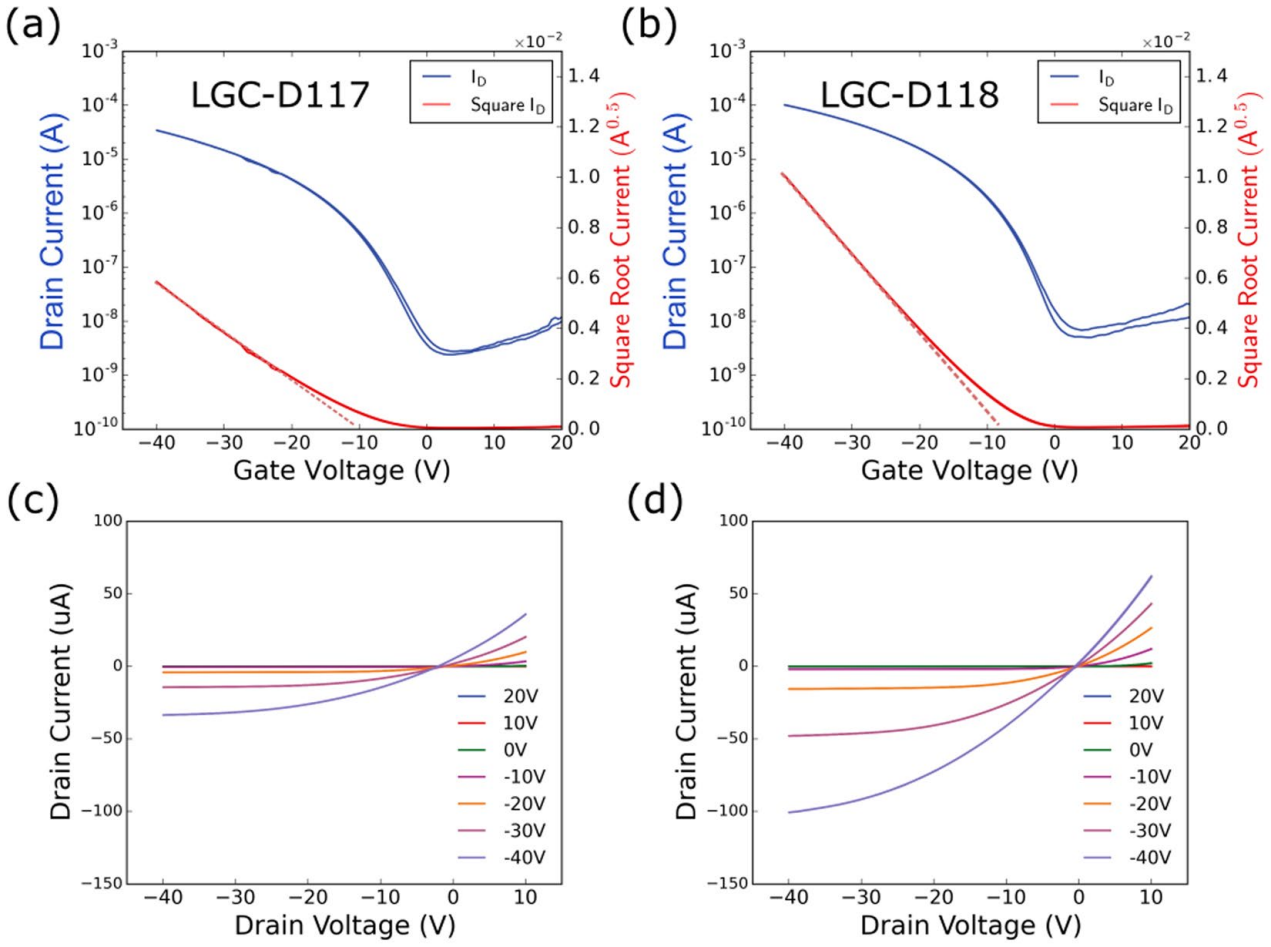
Typical transfer and output curves of organic field-effect transistors based on LGC-D117 and LGC-D118 OSCs after annealing at 100 °C. (V_sd_ = −40 V).

The charge carrier mobility (*μ*_FET_) in OFETs shows a strong dependence on the transfer integrals, which are sensitive to the π-π stacking distance and molecular packing conformation^24^. To investigate and compare the OFET properties of LGC-D117 and LGC-D118, top gate/bottom contact (TG/BC) transistors were fabricated. Each OSCs was dissolved in chlorobenzene (CB) at a concentration of 3 mg/mL, and the obtained solutions were spin-coated onto glass substrates with pre-patterned electrodes, which were used with and without annealing. Subsequently, CYTOP^TM^ was spin-coated onto the OSCs as a dielectric layer and annealed at 90 °C. All devices were characterized under N_2_ atmosphere in a glovebox, showing good linear and saturation regimes. Figure 3 shows typical transfer and output curves of OFETs based on LGC-D117 and LGC-D118, with their performance summarized in Table 2 (transfer curves of OFETs after annealing are shown in Figs S3 and S4). The transistor parameters, including *μ*_FET_, threshold voltage (*V*_th_), and the on/off ratio were calculated in the saturation regime using the standard OFET formula^44^. For LGC-D117, the spin-coated film exhibited a lowest mobility of 0.41 cm^2^ V^−1^ s^−1^ on average after annealing at 100 °C. Devices based on LGC-D117 annealed at higher temperatures show slightly higher performance (Fig. S3 and Table 2). In contrast, spin-coated LGC-D118 film exhibited average mobility of 1.76 cm^2^ V^−1^ s^−1^. For LGC-D118, having a solubilizing siloxane-terminated linear alkyl chain, the OFETs exhibited average mobilities of 1.76, 1.42, and 1.04 cm^2^ V^−1^ s^−1^ after annealing at 100, 120, and 140 °C, respectively, and highest mobility of 3.04 cm^2^ V^−1^ s^−1^ at 100 °C. Thus, the mobility increased with lower temperature annealing.

**Table 2:** Summary of devices incorporating different materials and annealing conditions.

Material/solvent	Annealing Temperature (°C)	Average Mobility (cm^2^ V^−1^ s^−1^)*	Highest Mobility (cm^2^ V^−1^ s^−1^)	Subthreshold Swing (V Dec^−1^)	Threshold Voltage (V)	On/Off Ratio (10^6^)	Contact Resistance (MΩ)**
LGC-D117/CB	100	0.41 ± 0.02	0.47	4.03 ± 0.07	−10.1 ± 0.88	1.38 ± 0.90	0.92 ± 0.03
	120	0.45 ± 0.03	0.52	4.42 ± 0.24	−10.4 ± 0.64	0.19 ± 0.09	0.80 ± 0.31
	140	0.52 ± 0.09	0.69	4.61 ± 0.38	−11.27 ± 1.14	0.71 ± 0.68	0.79 ± 0.28
LGC-D118/CB	100	1.76 ± 0.95	3.04	3.6 ± 0.50	−8.13 ± 1.49	1.00 ± 0.61	0.35 ± 0.14
	120	1.42 ± 0.43	2.18	3.8 ± 1.07	−4.62 ± 1.56	0.24 ± 0.17	0.21 ± 0.10
	140	1.04 ± 0.43	1.68	3.33 ± 0.83	−8.42 ± 2.62	0.46 ± 0.30	0.53 ± 0.48
LGC-D118/M-THF	100	1.53 ± 0.71	2.60	3.41 ± 1.27	−5.11 ± 1.58	0.82 ± 0.49	0.41 ± 0.19

*Error in average mobility = standard deviation (*σ*) of values. Averaging was performed for 4–8 devices.

**Contact resistance at channel width of 1000 µm obtained using the Y-function method^52^.

Environmentally friendly manufacturing processes are valuable for the commercialization of solution-processed OFETs, attracting much interest to the synthesis of high-performance semiconducting materials soluble in environmentally friendly solvents^45, 46^. Herein, we used the eco-friendly 2-methyltetrahydrofuran (M-THF) as M-THF solvent^47, 48^ for device fabrication. LGC-D118 was readily soluble in M-THF, and OFET fabricated using the corresponding M-THF solutions showed stable *p*-type operation (Fig. S6). In particular, LGC-D118 OFETs exhibited an average mobility of 1.53 cm^2^ V^−1^ s^−1^ and highest mobility of 2.60 cm^2^ V^−1^ s^−1^ after annealing at 100 °C as shown in Table 2.

## Discussion

The electronic wavefunction overlap determining the charge transfer integral is a sensitive function of precise molecular packing^49^. Therefore, we investigated the crystallinity of OSCs, which is important for understanding the high *μ*_FET_ of LGC-D118 OFETs, as the high mobility of LGC-D118 likely due to the higher crystallinity of their films. Polarization microscopy was utilized to investigate the crystalline morphologies of the fabricated thin films. Figure S7 shows polarized images of different as-spun films (images of annealed films are shown in Fig. S8), with different materials showing various morphologies. Highly crystalline domains commonly show strong birefringence under cross-polarized light, which confirms the crystalline nature of organic films. The LGC-D117 film exhibited a small domain size, in contrast to the LGC-D118 film. For the LGC-D118 film, the introduction of bulky siloxane-terminated hybrid alkyl chain increased the domain size. The crystal grain sizes of LGC-D118 were of the order of micrometers. These findings confirm the results of Bao *et al*., who reported that using an unconventional siloxane-terminated hexyl chain resulted in a closer backbone packing of the fabricated polymer^42^.

**Figure 4:**
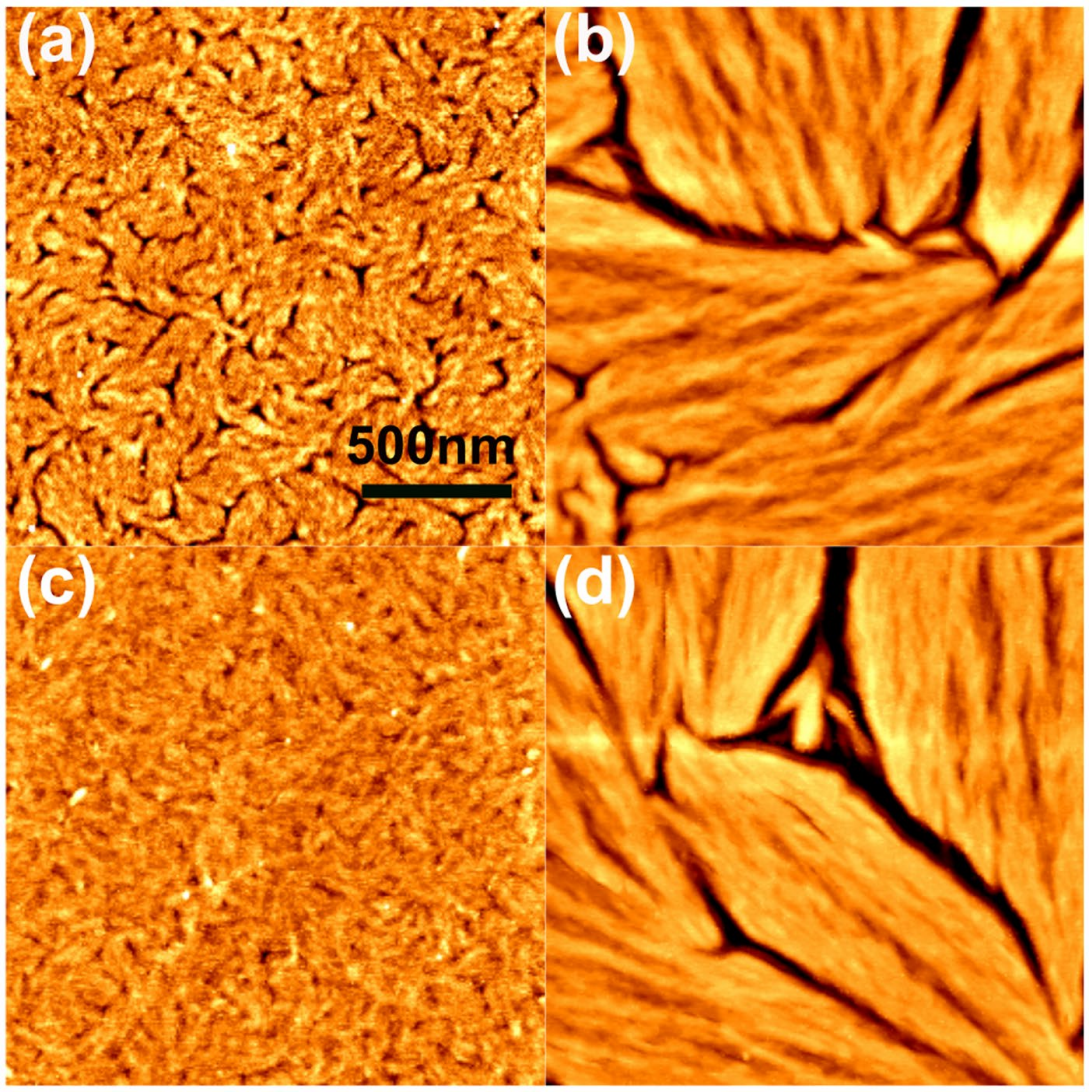
Atomic force microscopy imaging of the as-spun organic films for (**a**) LGC-D117, (**b**) LGC-D118 and of films annealed at 140 °C for (**c**) LGC-D117, (**d**) LGC-D118.

Atomic force microscopy (AFM) imaging of OSC films was further performed to confirm their microstructure. Figures 4 and S9 show the topography of LGC-D117 and LGC-D118 films, with a textured structure formed for all materials. In each domain, the microstructures are aligned parallel to one direction. We found that the surface of the LGC-D117 film featured small domains, with slight morphology changes observed after annealing at high temperatures. Compared to LGC-D117, AFM imaging of LGC-D118 showed a different morphology, the domain size being very large even for the as-spun film. However, the LGC-D118 film showed a larger grain boundary, and the gap between the disordered grains increased after thermal annealing, creating trapping sites for limiting *μ*_FET_ by charge transport at high annealing temperature (140 °C, Fig. 4(d)). This microstructure data can explain the mobility trend observed for different annealing temperatures.

**Figure 5:**
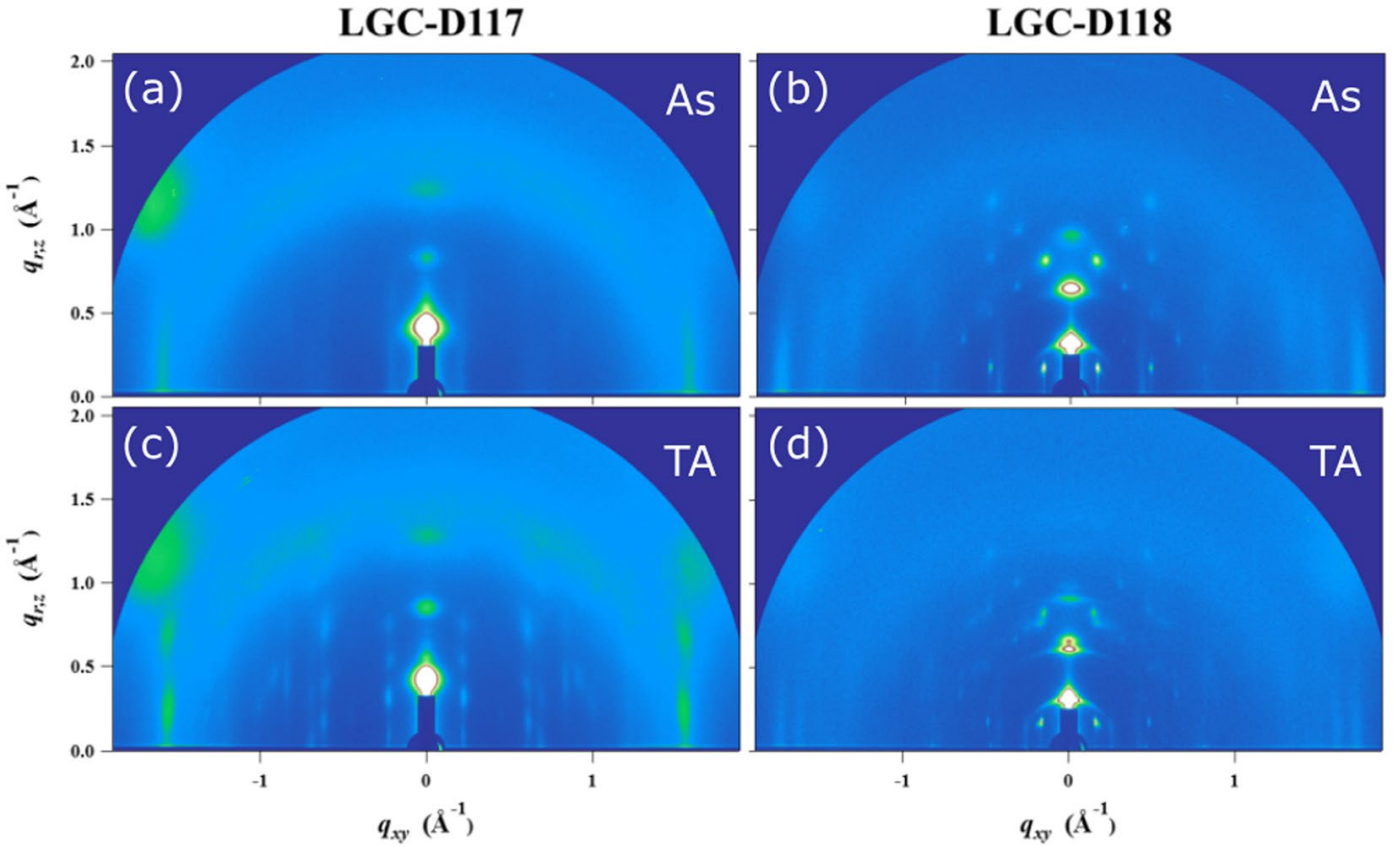
Grazing incidence X-ray diffraction patterns of the as-spun and thermally annealed (140 °C) LGC-D117 [(**a**) and (**c**)], LGC-D118 [(**b**) and (**d**)].

Two-dimensional (2D) grazing incidence X-ray diffraction (GIXRD) was used to analyze molecular packing in thin films (Figs 5 and S10), with the results summarized in Table S1. GIXRD data of all as-spun and annealed (140 °C) films showed strong edge-on orientation of molecules, with three visible orders of lamellar stacking peaks in the out-of-plane direction. The largest out-of-plane peak intensity was recorded for the as-spun LGC-D118 film (Table S1). Materials with different side chains exhibited different out-of-plane *d*-spacings. Compared to LGC-D117, which have alkyl side chains in their DPP units, LGC-D118 exhibited the longest *d*-spacing of 18.04 Å due to its siloxane-terminated solubilizing groups. The reason might be due to difference of intermolecular stacking distance and the side-chain ordering among those molecules^50^. The siloxane side chains in LGC-D118 also account for its high crystallinity (even for the as-spun film), being involved in strong intermolecular interactions^51^. The as-spun LGC-D118 film showed high crystallinity, which slightly decreased after annealing at 140 °C. The results of GIXRD analysis were strongly correlated with those of polarizing optical microscopy, AFM and OFET tests. LGC-D118 showed the highest mobility for as-spun samples, while OTFTs after annealing with increased temperature showed a lower performance. This crystallinity and mobility trend is rationalized by the properties of the employed side chains.

In conclusion, we report new D-A type DPP-based small molecules for high-performance OFETs. The highest *μ*_FET_ of 3.04 cm^2^ V^−1^ s^−1^ and excellent solubility were achieved for LGC-D118 OFETs due to the strong interaction between introduced siloxane side chains. Additionally, LGC-D118 was readily soluble in an environmentally friendly solvent (Fig. S12), sustaining its high mobility. GIXRD, AFM, and polarization microscopy results indicated that this remarkably high mobility could be attributed to the higher crystallinity and shorter intermolecular distance achieved by mild annealing. Our results demonstrate that small molecule with siloxane side chain can be used to prepare OSCs for organic electronics, showing high crystallinity, sufficient solubility, and low temperature processing, and possibly further boosting device performance.

## Methods

### Characterization and Measurements

Materials were characterized by ^1^H NMR spectroscopy (Agilent DD1, 500 MHz). Differential scanning calorimetry (DSC) was performed using a TA Instrument Q20 differential scanning calorimeter at heating rate of 10 °C/min in a nitrogen atmosphere. UV-vis spectra were obtained with a Mecasys Optizen Pop spectrophotometer. Cyclic voltammetry (CV) experiments were performed with an AutoLab analyzer. All CV measurements were carried out in 0.1 M tetrabutylammoniumtetrafluoroborate (Bu4NBF4) in acetonitrile with platinum as the counter electrode, indium tin oxide (ITO) coated with a thin film as the working electrode, and Ag/Ag+ electrode as the reference electrode, at a scan rate of 100 mV/s. To estimate the polymer energy levels from the vacuum energy level, we used the ferrocene/ferrocenium (Fc/Fc+) redox couple as a calibration reference. The half-wave potential (E1/2) for oxidation of the Fc/Fc+ redox couple was assumed to be 4.8 eV, below the vacuum level.

### Film Characterizations

Optical images were obtained using an OLYMPUS polarized microscope. 2D GIXRD measurements of the thin film microstructure were performed at the 9A beamlines of the Pohang Accelerator Laboratory (PAL). 11.07 KeV photons with grazing angle 0.13° were directed onto the sample to produce 2D scattering patterns. The surface morphology measurements were performed using an atomic force microscopy (AFM) (NX10, Park systems).

### Device fabrication and Characterization

OFET devices were fabricated in a TG/BC structure. Glass substrates were employed, and a lithographed electrode (Au/Ni = 13 nm/3 nm) was used as the source and drain electrodes. The glass substrates were sequentially cleaned with acetone, DI water, and isopropanol, and oven dried at 110 °C for 1 h. After drying, the substrates were treated with UV/Ozone for 30 min and then moved into a N_2_ filled glovebox. The pristine organic semiconductor layer was spin coated onto glass substrate from the solution (3 mg/ml in CB) at 2000 rpm and annealed at different temperatures for 1 h. CYTOP was spin coated on to the organic semiconductor as the dielectric layer (1:1 diluted) at 1500 rpm and annealed at 90 °C for 1 h. Al (50 nm) was used for the gate electrode and was thermally evaporated under vacuum (~10^−6^ Torr). Electrical characterization was measured under nitrogen using a Keithley semiconductor parametric analyzer (Keithley 4200-SCS). Hole mobility (μ) was determined using I_ds_ = (WC_i_/2 L) × μ × (V_g_ − V_th_)^2^ in the saturation regime, where C_i_ is the capacitance measured from OFET structure device (Fig. S11), I_ds_ is the drain-source current, V_g_ is gate voltage, and V_th_ is the threshold voltage.

## Electronic supplementary material

supplementary information
