# Supplementary material for: High Performance Solution Processed Organic Field Effect Transistors with Novel Diketopyrrolopyrrole-Containing Small Molecules
Source: Sci Rep. 2017 Mar 13;7:164. doi: 10.1038/s41598-017-00277-7 (PMC5427974; doi:10.1038/s41598-017-00277-7)
Supplement: Supplementary file 1 — supplementary information [file 41598_2017_277_MOESM1_ESM.doc]

High Performance Solution Processed Organic Field Effect Transistors with Novel Diketopyrrolopyrrole-Containing Small Molecules

Bogyu Lim^2‡^, Huabin Sun^1‡^, Jaechol Lee^2^, and Yong-Young Noh^1*^

^1^Department of Energy and Materials Engineering, Dongguk University, 30 Pildong-ro, 1-gil, Jung-gu, Seoul 04620, Republic of Korea ^2^Future Technology Research Center, Corporate R&D, LG Chem Research Park, 188, Moonji-ro, Yuseong-gu, Daejeon 34122, Republic of Korea

^‡^B. Lim and H. Sun contributed equally to this work.

Correspondence and requests for materials should be addressed to Y. Y. Noh (yynoh@dongguk.edu)

**Method:**

*Materials:*

All starting materials and reagents were purchased from commercial supplies unless otherwise specified, and used without further purification.

*Synthesis and Characterization of LGC-D117, LGC-D118*

Preparation of Compound 1

A lithium diisopropylamide (LDA) solution in THF (2.0 M, 31.25 ml, 62.6 mmol) at ‑78°C was added dropwise to a solution of 3-bromothiophene (10g, 61.33 mmol) in 100 ml of THF. After the mixture was stirred for 1 h at ‑78^o^C, a white suspension was formed. Then 63 ml (63 mmol) of trimethyltin chloride solution in hexane (1.0 M) was added at ‑78°C. After this addition, the mixture was stirring at same temperature for an additional 1 h, and then the ice bath was removed and the reaction mixture was allowed to warm gradually to room temperature. The reaction mixture was extracted with dichloromethane, washed with water, and dried with anhydrous MgSO4. After volatiles removal, crude product was obtained as a yellow liquid and used without further purification.

**Yield:** 93.58% (18.7 g). ^1^H NMR (CDCl_3_, 500 MHz, [ppm]): *δ* 7.49 (d, 1H, *J* = 4.63 Hz), 7.122 (d, 2H, *J* = 4.63 Hz), 0.445 (s, 9H). GC-MS: m/z 325.8, calcd 325.88

Preparation of Compound 2

Compound 1 (18.7 g, 57,39 mmol) at 80°C was added dropwise to a solution of 1,4-dibromo-2,5-diiodo-benzene (12.19 g, 25 mmol) and Pd(pph_3_)_2_Cl_2_ (702 mg, 1 mmol) in 100 ml of DMF under nitrogen atmosphere. The mixture was stirred for 40 h at 80°C. The reaction mixture was then extracted with dichloromethane, washed with water, and the solvent removed. Recrystallization using dichloromethane/methanol produced a slightly grey powder.

**Yield:** 75.56% (10.54 g). ^1^H NMR (CDCl_3_, 500 MHz, [ppm]): *δ* 7.712 (s, 2H), 7.424 (d, 2H, *J* = 5.33 Hz), 7.095 (d, 2H, *J* = 5.33 Hz); GC-MS: m/z 557.9, calcd 557.94.

Preparation of Compound 3

A 19.5 ml (39 mmol) of LDA solution (2.0 M) at ‑78°C was added dropwise to a solution of compound 2 (9.48 g, 17 mmol) in 300 ml of THF under nitrogen atmosphere. The mixture was stirred for 1 h at ‑78^o^C, and then chlorotrimethylsilane (4.95 ml, 39 mmol) was added at ‑78^o^C. After stirring for 1 h, the ice bath was removed and the mixture was allowed to warm gradually to room temperature. The reaction mixture was extracted with dichloromethane, washed with water, dried with anhydrous MgSO4, and the solvent was removed. The crude product was recrystallized in 2-propanol to obtain a white powder.

**Yield:** 89.40% (10.67 g). ^1^H NMR (CDCl_3_, 500 MHz, [ppm]): *δ* 7.684 (s, 2H), 7.159 (s, 2H), 0.359 (s, 18H); MS (APCI): m/z 701.72, calcd 697.74.

Preparation of Compound 4

Compound 3 (5.00g, 7.12 mmol) and 170 ml of anhydrous THF were put into a flask and cooled to ‑78^o^C. Subsequently, 38.22 ml (65 mmol) of *t*-BuLi in pentane (1.7 M) was added dropwise and the reaction mixture was stirred for 2 h at the same temperature. bis(2-ethylhexyl)dichlorosilane was then added slowly, and the mixture allowed to warm to room temperature then stirred for 3 h. The mixture was poured into water, extracted by diethyl ether three times, and washed with water. The solvents were removed via rotary evaporation. Finally, the crude compound was purified by column chromatography with hexane eluent. The product was obtained as a viscous pale yellow oil.

**Yield:** 87.35 % (5.54 g). ^1^H NMR (CDCl_3_, 500 MHz, [ppm]): *δ* 7.570 (s, 2H), 7.197 (s, 2H), 1.407–1.211 (m, 44H), 0.930–0.851 (m, 24H), 0.348 (s, 18H); MS (APCI): m/z 891.4, calcd 890.56.

Preparation of Compound 5

Compound 4 (5.41 g, 6.07 mmol) and NBS (2.50 g, 14.07 mmol) were dissolved into 300 ml of THF in a round flask under nitrogen protection, the solution was protected from light and stirred at room temperature. After 12 h, the mixture was poured into 200 ml of water and extracted with diethyl ether. The volatiles were removed under vacuum, and the residue was purified by silica gel chromatography using hexane eluent. The product was obtained as a light yellow oil.

**Yield:** 94.32% (5.18 g). ^1^H NMR (CDCl_3_, 500 MHz, [ppm]): *δ* 7.428 (s, 2H), 7.033 (s, 2H), 1.385–0.969 (m, 44H), 0.814–0.743 (m, 24H); GCMS: m/z 906.26, calcd 902.30.

Preparation of Compound 6

*t*-BuLi solution (6.76 ml, 11.5 mmol) in pentane (1.7 M) was added dropwise to compound 5 (2.08 g, 2.30 mmol) in 100 ml of dry THF at ‑78°C. After complete addition, the mixture was stirred at ‑78°C for 0.5 h, and the reactant was stirred at room temperature for an additional 1 h. The mixture was cooled to ‑78°C, and 12 ml (12 mmol) of trimethyltinchloride solution in hexane (1.0 M) was added, and then allowed to warm to room temperature and stirred for 3 h. The mixture was poured into water and extracted by diethyl ether two times, the solvent was evaporated, and the crude product was extracted by hexane one more time. The organic layer was dried over anhydrous MgSO4 and decolorized with activated carbon. After volatiles removal the product was obtained as a green oil and used without further purification.

**Yield:** 82.61% (2.04 g). ^1^H NMR (CDCl_3_, 500 MHz, [ppm]): *δ* 7.564 (s, 2H), 7.140 (s, 2H), 1.448–0.956 (m, 44H), 0.794–0.748 (m, 24H), 0.380 (s, 18H).

Preparation of Compound 7

Et_3_N (3 ml, 21.5 mmol) and trihexylchlorosilane (7.46 ml, 20.37 mmol) at 0 °C was added to a CH_2_Cl_2_ (30 ml) solution of 2,5-dibromo-1,4-dihydroxybenzene (2.00 g, 7.47 mmol) and 4-(dimethylamino) pyridine (0.35 g, 2.92 mmol), and the mixture was stirred at room temperature for 12 h. After concentration, the residue was diluted with water and extracted with dichloromethane. The extracts were washed with brine, dried, and purified over silica gel with hexane eluent. The final product was a colorless oil.

**Yeild:** 94.17% (5.86 g). ^1^H NMR (CDCl_3_, 500 MHz, [ppm]): *δ* 6.994 (s, 2H), 1.368-1.270 (m, 48H), 0.893–0.867 (t, 18H, *J* = 13 Hz), 0.761–0.729 (t, 12H, *J* = 16 Hz); GCMS: m/z 832.47, calcd 830.41.

Preparation of Compound 8

2-tributylstannyl thiophene (9.33 g, 25 mmol), tris(dibenzylideneacetone)dipalladium(0) (0.458 g, 0.5 mmol), triphenylphosphine (0.52 g, 2 mmol) and 70 ml of degassed toluene were added to compound 7 (5.00 g, 6 mmol) in a 250 ml of flask. The mixture was stirred for 40 h at 110°C. After cooling, the mixture was extracted with dichloromethane, washed with water, and the solvent was removed. The residue was purified over silica gel with hexane eluent and produced a yellow oil.

**Yeild:** in 59.16% (2.98 g). ^1^H NMR (CDCl_3_, 500 MHz, [ppm]): *δ* 7.409 (d, 2H *J* = 3 Hz), 7.313 (d, 2H, *J* = 5 Hz), 7.136 (s, 2H), 7.085 (t, 2H, *J* = 8.5 Hz), 1.343–1.237 (m, 48H), 0.871–0.844 (t, 18H, *J* = 13.5 Hz), 0.784–0.751 (t, 12H, *J* = 16.5 Hz); GCMS: m/z 839.72, calcd 838.56.

Preparation of Compound 9

5.325 ml (10.65 mmol) of lithium diisopropylamide (LDA) solution in THF (2.0 M) was added dropwise to compound 8 (2.98 g, 3.55 mmol) in 100 ml of dry THF at –78°C. The mixture was then stirred at –78°C for 2 h, and 11 ml (11 mmol) of trimethyltinchloride solution in hexane (1.0 M) was added, the reaction allowed to warm to room temperature, and stirred for 3 h. The mixture was poured into water and extracted by dichloromethane two times, and the solvent evaporated. The crude product was extracted by hexane one more time. The organic layer was dried over anhydrous MgSO4 and decolorized with activated carbon. After volatiles removal, the product was obtained as a yellow oil and used without further purification.

**Yield:** 83.41% (3.45 g). ^1^H NMR (CDCl_3_, 500 MHz, [ppm]): *δ* 7.517 (s, 2H), 7.154 (m, 4H), 1.340–1.236 (m, 48H), 0.869–0.843 (t, 18H, *J* = 13 Hz), 0.780–0.749 (t, 12H, *J* = 15.5 Hz); GCMS: m/z 1164.5, calcd 1166.49.

Preparation of Compound 10

2,5-Di(2-ethylhexyl)-3,6-di(thiophen-2-yl)-diketopyrrolopyrroleexyl (2.62 g, 5.00 mmol) and NBS (0.907 g, 5.1 mmol) were dissolved into 70 ml of CHCl_3_ in a round flask under N_2_ protection, The solution was protected from light and stirred at room temperature for 48 h. The mixture was then poured into 50 ml of water and extracted with dichloromethane. Volatiles were removed under vacuum, and the residue was purified by silica gel chromatography using hexane to 50% dichloromethane eluent. The final product was a dark black powder.

**Yeild:** 40% (1.2 g). ^1^H NMR (CDCl_3_, 500 MHz, [ppm]): *δ* 8.908 (d, 1H, *J*= 5 Hz), 8.636 (d, 1H, *J* = 4.5 Hz), 7.649(d, 1H, *J* = 6 Hz), 7.282 (t, 1H, *J* = 8.5 Hz), 7.228 (d, 1H, *J* = 4.5 Hz), 4.033–3.926 (m, 4H), 1.854 (br, 2H), 1.360–1.242 (m, 16H), 0.888–0.852 (m, 12H)

Preparation of Compound 11

3,6-Dithien-2-yl-2,5-bis[6-(1,1,1,3,5,5,5,-heptamethyltrisiloxan-3-yl)hexyl]-pyrrolo[3,4-

c]pyrrole-1,4-dione. (7.93 g, 9.00 mmol) and NBS (1.92 g, 10.8 mmol) were dissolved into 180 ml of CHCl_3_ in a round flask under N_2_ protection, The solution was protected from light and stirred at room temperature for 48 h. The mixture was then poured into 250 ml of water and extracted with dichloromethane. Volatiles were removed under vacuum, and the residue was purified by silica gel chromatography using hexane to 50% dichloromethane eluent. The final product was a dark black powder.

**Yield:** 32% (3.13 g). ^1^H NMR (CDCl_3_, 500 MHz, [ppm]): *δ* 8.948 (d, 1H, *J*= 6 Hz), 8.681 (d, 1H, *J* = 4 Hz), 7.654 (d, 1H, *J* = 6 Hz), 7.296 (t, 1H, *J* = 8.5 Hz), 7.243 (d, 1H, *J* = 4 Hz), 4.078 (t, 2H, *J* = 16 Hz), 4.009 (t, 2H, *J* = 15.5 Hz), 1.738 (br, 4H), 1.467–1.368 (m, 8H), 0.487–0.455 (m, 4H), 0.083–0.072 (br, 36H), ‑0.009 (br, 6H); MALDI-TOF: m/z 958.30, calcd 958.22.

Preparation of Compound 12:

Compound 10 (6.03 g, 10 mmol), 5-formyl-2-thienylboronic acid (2.65 g, 17 mmol), tetrakis(triphenylphosphine)palladium(0) (Pd(pph_3_)_4_, 0.35 g, 0.3 mmol), 75 ml of 2 M K2CO3, and 300 ml of THF were added in a 1000 ml flask and the mixture refluxed for 40 h. After cooling, the mixture was extracted with dichloromethane three times, washed with water, and solvent removed. The residue was purified over silica gel with hexane:dichloromethane ratio 1:1–1:2 gradually as eluent and gave product as a dark purple solid.

**Yeild:** 87.3% (5.542 g).^1^H NMR (CDCl_3_, 500 MHz, [ppm]): *δ* 9.903 (s, 1H), 8.951 (d, 1H, *J*= 5 Hz), 8.882 (d, 1H, *J* = 4 Hz), 7.726 (d, 1H, *J* = 4 Hz), 7.667 (t, 1H, *J* = 6 Hz), 7.485 (d, 1H, *J* = 4 Hz), 7.388 (d, 1H, *J* = 4 Hz), 7.297 (t, 1H, *J* = 9 Hz), 4.054–4.035 (m, 4H), 1.900 (br, 2H), 1.368–1.257 (m, 16H), 0.913–0.858 (m, 12H); MS (APCI): m/z 635.10, calcd 634.24.

Preparation of Compound 13:

Compound 11 (4.09 g, 4.26 mmol), 5-formyl-2-thienylboronic acid (0.935 g, 6 mmol), tetrakis(triphenylphosphine)palladium(0) (Pd(pph_3_)_4_, 0.24 g, 0.21 mmol), 50 ml of 2 M K2CO3, and 200 ml of THF were added in a 500 ml flask and the mixture refluxed for 40 h. After cooling, the mixture was extracted with dichloromethane three times, washed with water, and solvent removed. The residue was purified over silica gel with hexane:dichloromethane ratio 10:1–1:1 gradually as eluent and gave product as a dark purple solid.

**Yield:** 79% (3.33 g). ^1^H NMR (CDCl_3_, 500 MHz, [ppm]): *δ* 9.888 (s, 1H), 8.987 (d, 1H, *J*= 5 Hz), 8.884 (d, 1H, *J* = 4 Hz), 7.705 (d, 1H, *J* = 4 Hz), 7.658 (d, 1H, *J* = 6 Hz), 7.467 (d, 1H, *J* = 4 Hz), 7.385 (d, 1H, *J* = 3.5 Hz), 7.293 (t, 1H, *J* = 9 Hz), 4.089 (t, 4H, *J* = 15.5 Hz), 1.758 (br, 4H), 1.463–1.380 (m, 8H), 0.490–0.477 (m, 4H), 0.079–0.067 (br, 36H), -0.008 (br, 6H); MS (APCI): m/z 991.40, calcd 990.30.

Preparation of Compound 14:

Compound 12 (5.54 g, 8.73 mmol) and NBS (1.865 g, 10.48 mmol) were dissolved into 70 ml of CHCl_3_ in a round flask under N_2_ protection. The solution was protected from light and stirred at room temperature for 48 h, then poured into 100 ml of water and extracted with dichloromethane. Volatiles were removed under vacuum, and the residue was purified by silica gel chromatography using chloroform:hexane 1:10–1:1 gradually as eluent and produced a dark purple powder.

**Yeild:** 71.5% (4.45 g).^1^H NMR (CDCl_3_, 500 MHz, [ppm]): *δ* 9.905 (s, 1H), 8.893 (d, 1H, *J*= 4 Hz), 8.691 (d, 1H, *J* = 4 Hz), 7.725 (d, 1H, *J* = 4 Hz), 7.483 (t, 1H, *J* = 4 Hz), 7.391 (d, 1H, *J* = 4 Hz), 7.244 (d, 1H, *J* = 4.5 Hz), 4.040 (t, 2H, *J* = 14.5 Hz), 3.971 (t, 4H, *J* = 15 Hz), 1.891 (br, 2H), 1.376–1.255 (m, 16H), 0.912–0.897 (m, 12H); MS (APCI): m/z 715.10, calcd 712.15.

Preparation of Compound 15:

Compound 13 (3.00 g, 3.03 mmol) and NBS (0.59 g, 3.33 mmol) were dissolved into 80 ml of CHCl_3_ in a round flask under N_2_ protection. The solution was protected from light and stirred at room temperature for 48 h, then poured into 100 ml of water and extracted with dichloromethane. Volatiles were removed under vacuum, and the residue was purified by silica gel chromatography using hexane: dichloromethane 10:1–1:1 gradually as eluent and produced a dark purple powder.

**Yield:** 81% (2.63 g). ^1^H NMR (CDCl_3_, 500 MHz, [ppm]): *δ* 9.905 (s, 1H), 8.896 (d, 1H, *J*= 4 Hz), 8.732 (d, 1H, *J* = 4.5 Hz), 7.720 (d, 1H, *J* = 3.5 Hz), 7.492 (d, 1H, *J* = 4 Hz), 7.405 (d, 1H, *J* = 4 Hz), 7.249 (d, 1H, *J* = 5 Hz), 4.088 (t, 2H, *J* = 15.5 Hz), 4.018 (t, 2H, *J* = 15.5 Hz), 1.754 (br, 4H), 1.456–1.378 (m, 8H), 0.491–0.459 (m, 4H), 0.078–0.063 (br, 36H), -0.017 (s, 6H); MS (APCI): m/z 1071.0, calcd 1068.21.

Preparation of Compound 16

Compound 6 (0.858 g, 0.8 mmol), compound 14 (1.342 g, 1.88 mmol), Pd_2_dba_3_(36 mg, 0.04 mmol), PPh_3_ (42 mg, 0.16 mmol), toluene (60 ml) and 6 ml of DMF were added and the mixture heated to 110^o^C for 48 h. After cooling, the residue was precipitated in 200 ml MeOH, then filtered. The filtered compound was dissolved in chloroform and washed with water. After volatiles were removed, the crude was purified by flash chromatography using dichloromethane and then chloroform eluents. The dark brown solid product was recrystallized with dichloromethane/MeOH and filtered. The solid was washed with copious methanol, and dried under vacuum for 24 h. The product collected was a dark purple powder.

**Yield:** 58% (0.93 g). ^1^H NMR (CDCl_3_, 500 MHz, [ppm]): *δ* 9.903 (s, 2H), 9.078 (d, 2H, *J*= 4 Hz), 8.878 (d, 2H, *J* = 3.5 Hz), 7.727 (d, 2H, *J* = 3.5 Hz),7.592 (s, 2H), 7.489 (d, 2H, *J* = 4 Hz), 7.387 (d, 2H, *J* = 3 Hz), 7.356 (br, 4H), 4.076 (br, 8H), 1.969 (br, 4H), 1.399–1.160 (br, 76H), 0.941–0.797 (m, 48H); MALDI-TOF: m/z 2011.2, calcd 2010.92.

Preparation of Compound 17

Compound 6 (1.18 g, 1.1 mmol), compound 15 (2.62 g, 2.45 mmol), Pd_2_dba_3_(50 mg, 0.055 mmol), PPh_3_ (58 mg, 0.22 mmol), toluene (60 ml) and 6 ml of DMF were added and the mixture heated to 110^o^C for 48 h. After cooling, the residue was precipitated in 200 ml MeOH, then filtered. The filtered compound was dissolved in chloroform and washed with water. After volatiles were removed, the crude was purified by flash chromatography using dichloromethane and then chloroform eluents. The dark brown solid product was recrystallized with dichloromethane/MeOH and filtered. The solid was washed with copious methanol, and dried under vacuum for 24 h. The product collected was a dark purple powder.

**Yield**: 57% (1.72 g). ^1^H NMR (CDCl_3_, 500 MHz, [ppm]): *δ* 9.905 (s, 2H), 9.058 (d, 2H, *J*= 4.5 Hz), 8.885 (d, 2H, *J* = 4 Hz), 7.724 (d, 2H, *J* = 3.5 Hz), 7.600 (s, 2H), 7.499 (d, 2H, *J* = 3.5 Hz), 7.402 (d, 2H, *J* = 2.5 Hz), 7.380 (br, 4H), 4.125 (br, 8H), 1.796 (br, 8H), 1.545-1.034 (m, 60H),0.808 (br, 24H), 0.507 (br, 8H), 0.071 (br, 72H), ‑0.002 (br, 12H); MALDI-TOF: m/z 2723.7, calcd 2723.04.

Preparation of Compound LGC-D117

Under the protection of a nitrogen, three drops of piperidine was added to compound 16 (0.402 g, 0.2 mmol) and 3-octylrhodanine (0.490 g, 2 mmol) in a solution of 20 ml of dry CHCl_3_. The resulting solution was refluxed and stirred for 72 h under nitrogen. The mixture was then extracted with CH_2_Cl_2_, washed with water, and dried over MgSO_4_. After solvent removal, it was recrystallized with CH_2_Cl_2_/MeOH and then purified by chromatography on a silica gel column using CH_2_Cl_2_ then chloroform eluents. The resulting dark purple solid was recrystallized with chloroform/acetone and filtered. The solid was washed with copious methanol, and dried under vacuum for 24 h. LGC-D117 was a dark purple solid.

**Yield:** 66% (326 mg). ^1^H NMR (CDCl_3_, 500 MHz, [ppm]): *δ* 9.073 (d, 2H, *J*= 4 Hz), 8.912 (d, 2H, *J* = 4 Hz), 7.831 (s, 2H), 7.594 (s, 2H), 7.447 (d, 2H, *J* = 4 Hz), 7.370–7.348 (m, 8H), 4.128 (br, 12H), 1.977 (m, 4H), 1.714 (m, 4H), 1.420–1.165 (br, 96H), 0.948–0.800 (m, 54H); MALDI-TOF: m/z 2065.3, calcd 2465.08.

Preparation of Compound LGC-D118

Under the protection of a nitrogen, three drops of piperidine was added to compound 17 (0.409 g, 0.15 mmol) and 3-octylrhodanine (0.37 g, 1.5 mmol) in a solution of 20 ml of dry CHCl_3_. The resulting solution was refluxed and stirred for 72 h under nitrogen. The mixture was then extracted with CH_2_Cl_2_, washed with water, and dried over MgSO_4_. After solvent removal, it was recrystallized with CH_2_Cl_2_/MeOH and then purified by chromatography on a silica gel column using CH_2_Cl_2_ then chloroform eluents. The resulting dark purple solid was recrystallized with chloroform/acetone and filtered. The solid was washed with copious methanol, and dried under vacuum for 24 h. LGC-D118 was a dark purple solid.

**Yield:** 67% (0.32 g). ^1^H NMR (CDCl_3_, 500 MHz, [ppm]): *δ* 9.062 (d, 2H, *J*= 3.5 Hz), 8.923 (d, 2H, *J* = 4.5 Hz), 7.836 (s, 2H), 7.605 (s, 2H), 7.459 (d, 2H, *J* = 4 Hz), 7.386 (m, 8H), 4.134 (br, 12H), 1.810 (br, 8H), 1.795 (br, 4H), 1.545–1.171 (m, 80H), 0.904–0.807 (br, 30H), 0.518 (br, 8H), 0.084 (br, 72H), ‑0.002 (br, 12H); MALDI-TOF: m/z 3177.5, calcd 3177.20.

*Film Characterization of Film.*

Optical images were obtained using an OLYMPUS polarized microscope. 2D GIXRD measurements of the thin film microstructure were performed at the 9A beamlines of the Pohang Accelerator Laboratory (PAL). 11.07 KeV photons with grazing angle 0.13^o^ were directed onto the sample to produce 2D scattering patterns.

## OFET Fabrication and Measurement

OFET devices were fabricated in a TG/BC structure. Glass substrates were employed, and a lithographed electrode (Au/Ni = 13 nm/3 nm) was used as the source and drain electrodes. The glass substrates were sequentially cleaned with acetone, DI water, and isopropanol, and oven dried at 110 °C for 1 h. After drying, the substrates were treated with UV/Ozone for 30 min and then moved into a N_2_ filled glovebox. The pristine organic semiconductor layer was spin coated onto glass substrate from the solution (3 mg/ml in CB) at 1500 rpm and annealed at different temperatures for 1h. CYTOP was spin coated on to the organic semiconductor as the dielectric layer (1:1 diluted) at 2000rpm and annealed at 90 °C for 1h. Al (50 nm) was used for the gate electrode and was thermally evaporated under vacuum (∼10^–6^ Torr). Electrical characterization was measured under nitrogen using a Keithley semiconductor parametric analyzer (Keithley 4200-SCS). Hole mobility (μ) was determined using I_ds_ = (WC_i_/2L) × μ × (V_g_ – V_th_)^2^ in the saturation regime, where C_i_ is the capacitance measured from OFETs (Fig. S11), I_ds_ is the drain-source current, V_g_ is gate voltage, and V_th_ is the threshold voltage.

**Figure S1**. Differential scanning calorimetry (DSC) curves of LGC-D117 and LGC-D118.

**Figure S2**. Cyclic voltammogram of LGC-D117, LGC-D118


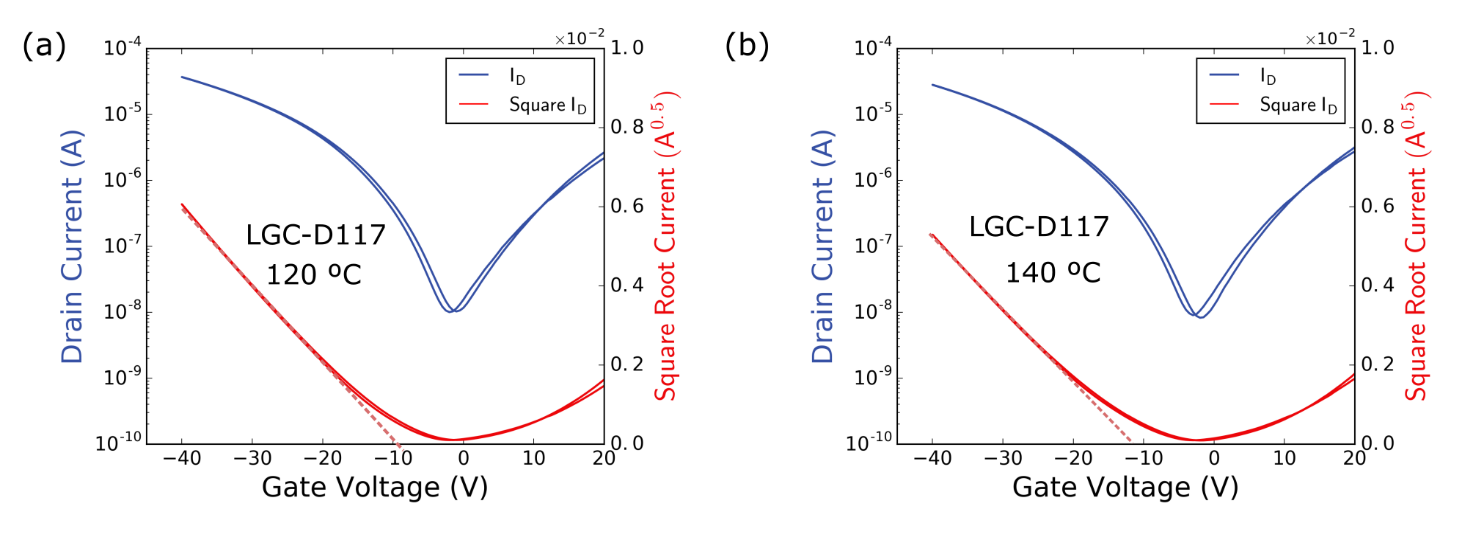


**Figure S3.** Typical transfer curves of organic field effect transistors based on LGC-D117, after annealing at 120 °C and 140 °C. (Drain Voltage is -40V)


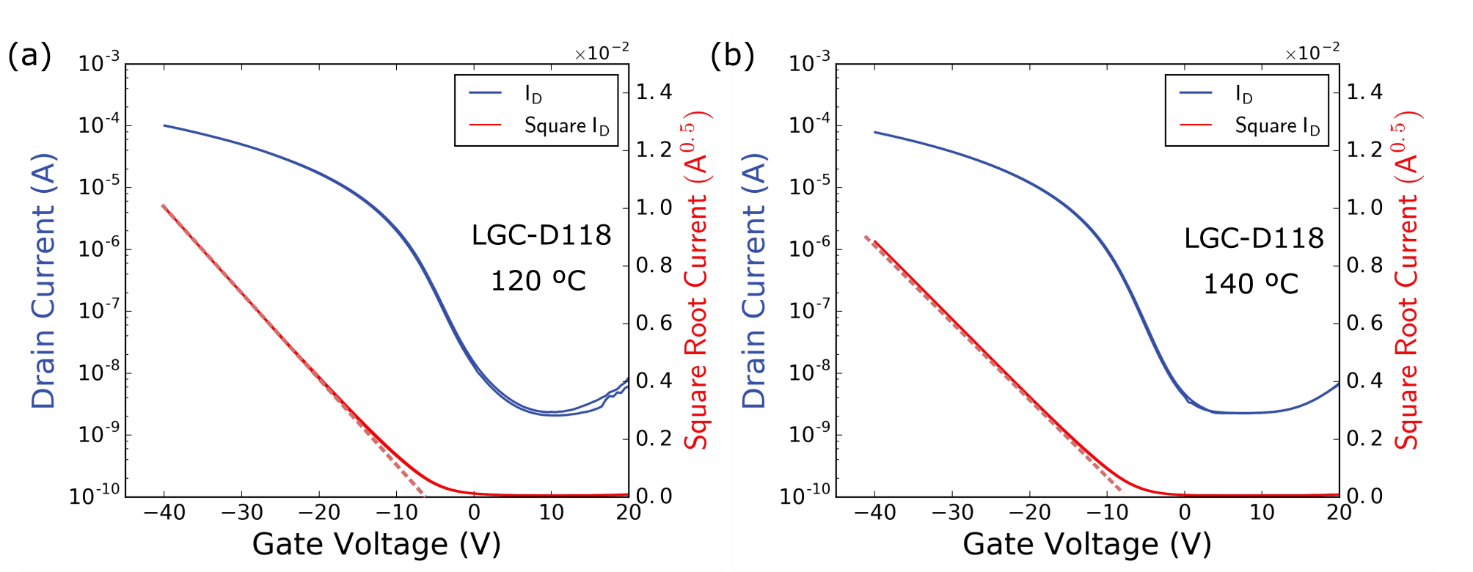


**Figure S4.** Typical transfer curves of organic field effect transistors based on LGC-D118 after annealing at 120 °C and 140 °C. (Drain Voltage is -40V).


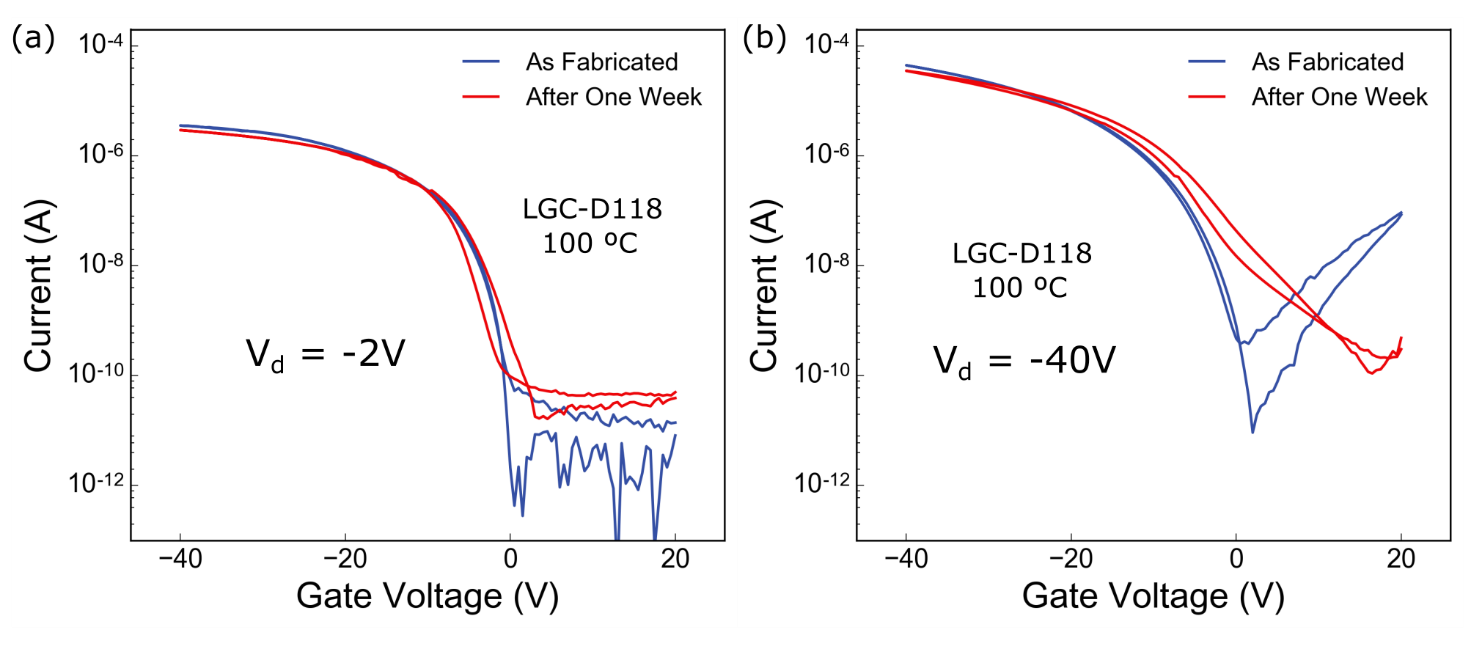


**Figure S5.** The device transfer curve of LGC-D118 as-fabricated and after putting in the air for one weeks, (a-b) Linear and saturation region of LGC-D118 based transistor before and after one week in air.


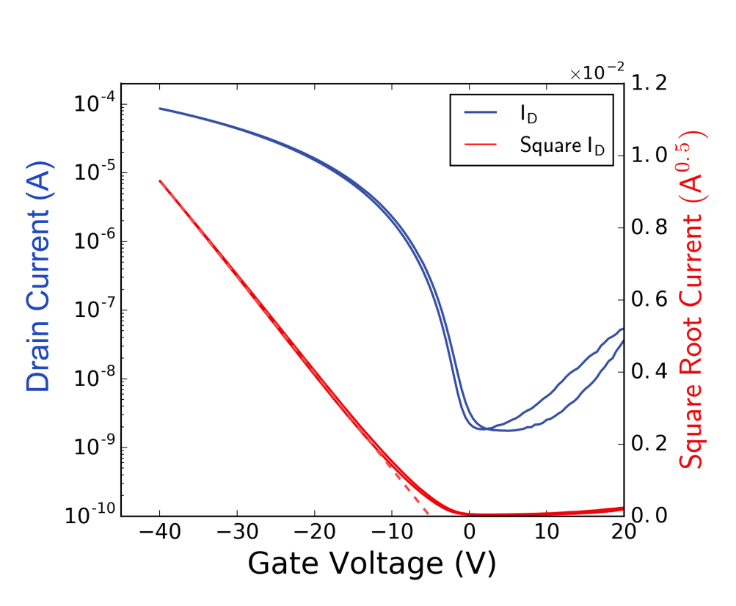


**Figure S6.** Typical transfer curves of organic field effect transistors based on LGC-D118 at 100 °C from eco-friendly solvent 2-methyltetrahydrofuran. (Drain Voltage is -40V).


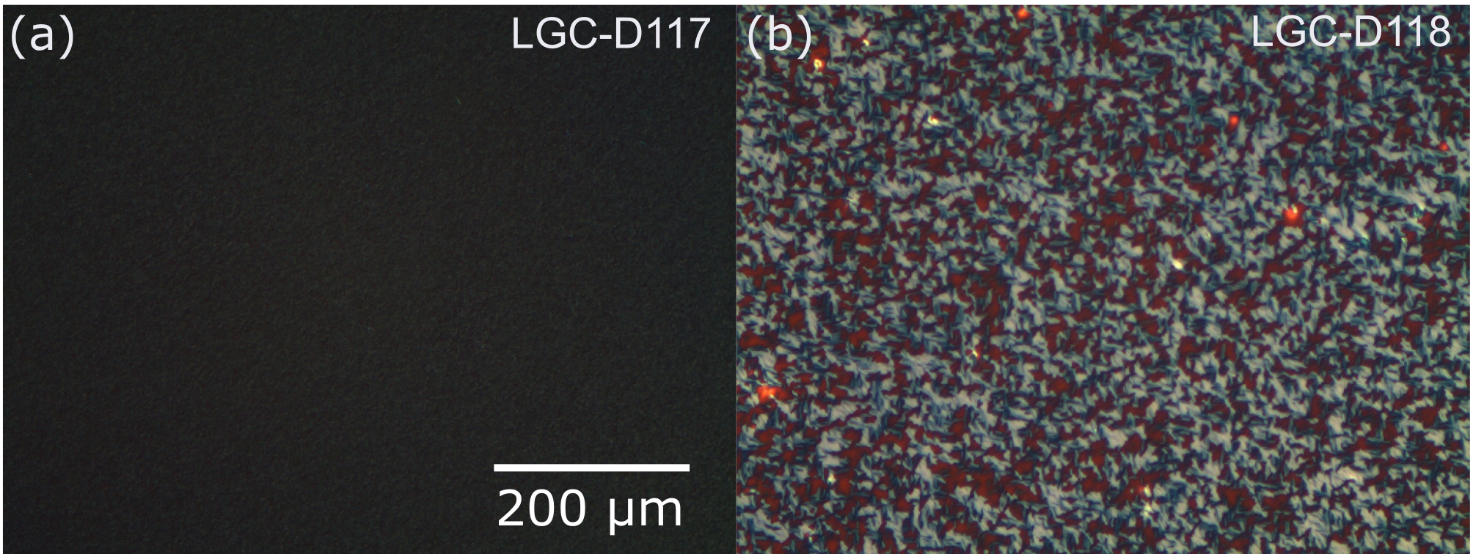


**Figure S7.** Polarized optical images of as-spun organic films for (a) LGC-D117, (b) LGC-D118.


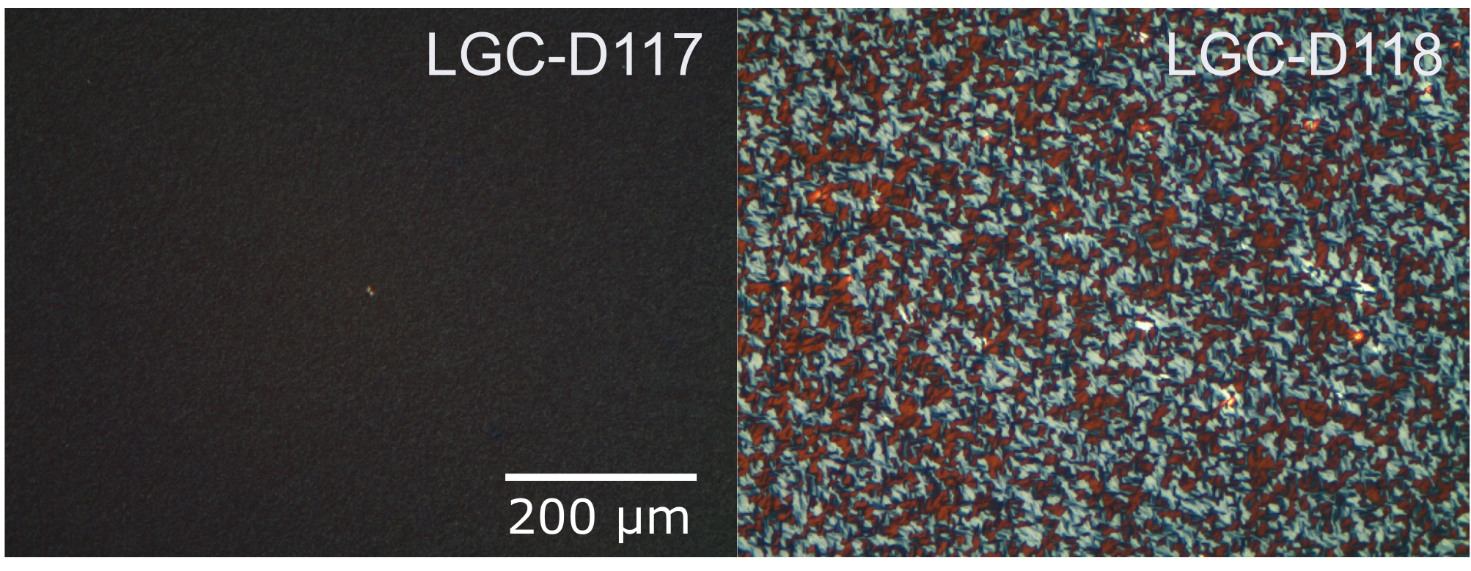


**Figure S8.** Polarized microscope images of 140 °C thermal annealed LGC-D117, LGC-D118 films.


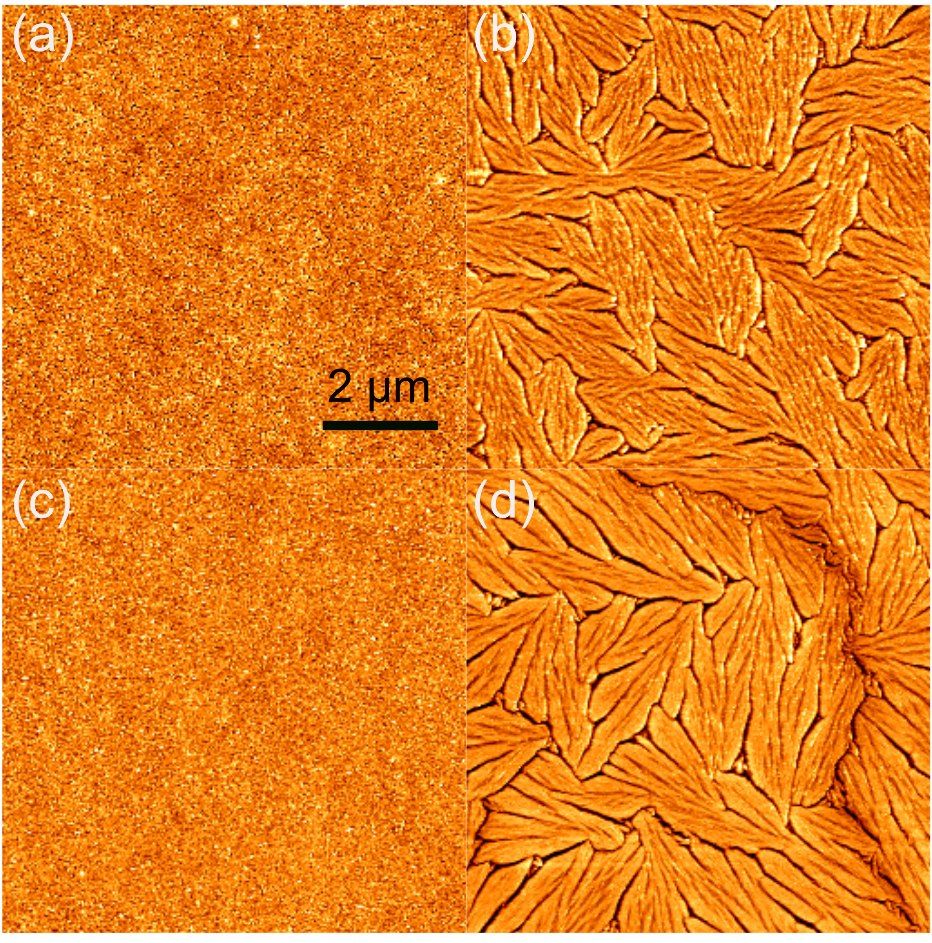


**Figure S9.** Large Scale Atomic Force Microscope images of as-spun organic films for (a) LGC-D117, (b) LGC-D118, and 140 °C annealed films for (c) LGC-D117, (d) LGC-D118.


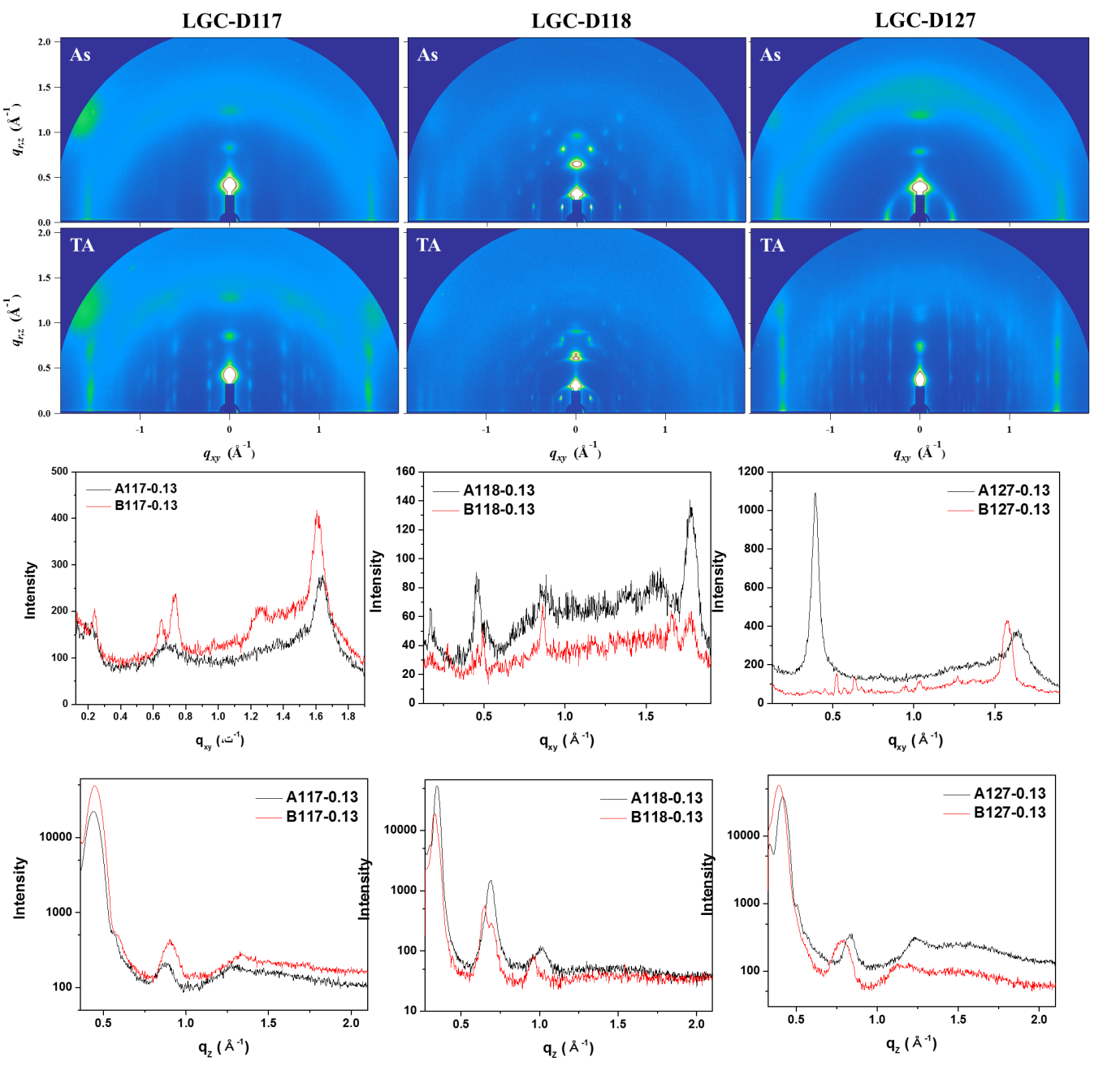


**Figure S10.** Grazing incidence X-ray diffraction patterns of as spun and 140 °C thermal annealed LGC-D117 [(a) & (c)], LGC-D118 [(b) & (d)] film.


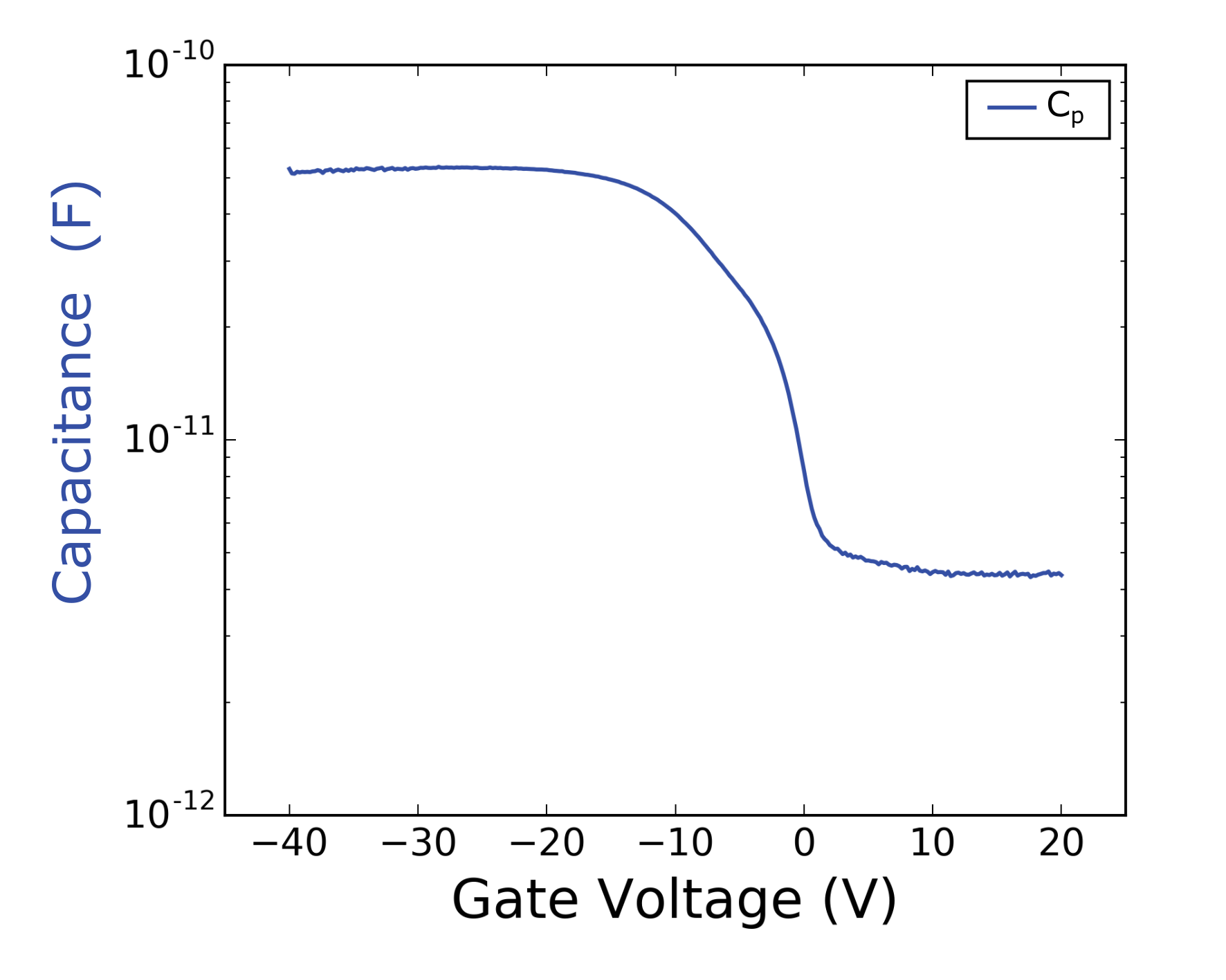


**Figure S11.** Gate voltage dependent Capacitance of Organic Field Effect Transistor at 100 Hz with same structure as we used for transfer curve measurement ( ~ 10 nF/cm^2^).


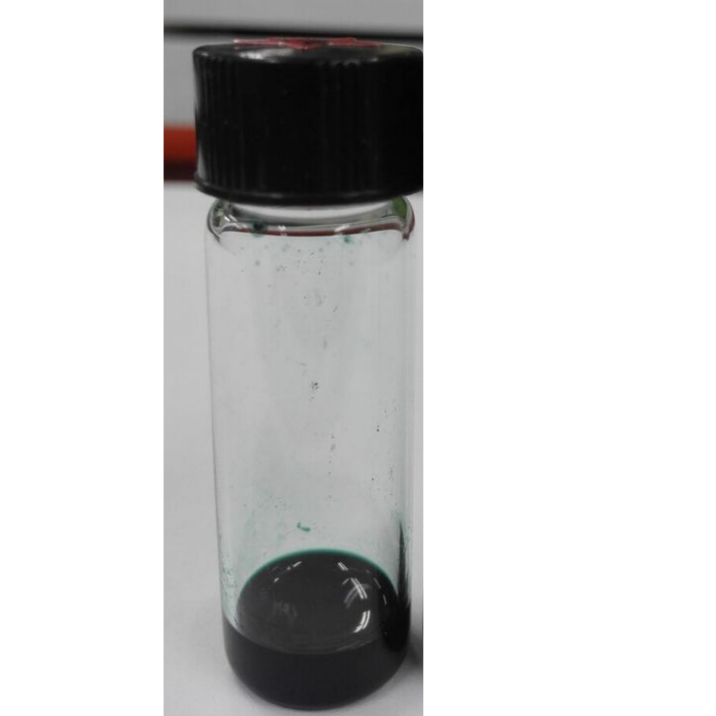


**Figure S12.** LGC-D118 dissolved in 2-methyltetrahydrofuran at concentration of 3mg/ml.

**Table S1.** Summary of grazing incidence X-ray diffraction data.

| Material | Peak Intensity | | | Q (nm^-1^) | | d (nm) |
| --- | --- | --- | --- | --- | --- | --- |
| LGC-D117  as-spun | Z plane | (100) | 22263 | Q_z_ | 0.44 | 14.34 |
|  |  | (200) | 219 |  | 0.88 | 7.18 |
|  |  | (300) | 216 |  | 1.28 | 4.92 |
|  | XY plane | | 178.5 | Q_xy_ | 0.22 | 28.68 |
|  |  |  | 137.5 |  | 0.68 | 9.29 |
|  |  |  | 278 |  | 1.64 | 3.82 |
| LGC-D117 annealed  (140 °C) | Z plane | (100) | 48801 | Q_z_ | 0.45 | 14.09 |
|  |  | (200) | 441 |  | 0.90 | 6.95 |
|  |  | (300) | 302 |  | 1.33 | 4.73 |
|  | XY plane | | 207 | Q_xy_ | 0.24 | 26.27 |
|  |  |  | 183 |  | 0.65 | 9.67 |
|  |  |  | 239 |  | 0.74 | 8.52 |
|  |  |  | 213 |  | 1.29 | 4.85 |
|  |  |  | 418 |  | 1.60 | 3.91 |
| LGC-D118  as-spun | Z plane | (100) | 55774 | Q_z_ | 0.35 | 18.04 |
|  |  | (200) | 1485 |  | 0.69 | 9.09 |
|  |  | (300) | 122 |  | 1.01 | 6.25 |
|  | XY plane | | 90.5 | Q_xy_ | 0.45 | 13.86 |
|  |  |  | 89 |  | 0.88 | 7.12 |
|  |  |  | 140.5 |  | 1.77 | 3.54 |
| LGC-D118 annealed  (140 °C) | Z plane | (100) | 18733 | Q_z_ | 0.33 | 18.80 |
|  |  | (200) | 565 |  | 0.65 | 9.66 |
|  |  | (300) | 82 |  | 0.96 | 6.56 |
|  | XY plane | | 52.5 | Q_xy_ | 0.49 | 12.79 |
|  |  |  | 69 |  | 0.86 | 7.30 |
|  |  |  | 64 |  | 1.65 | 3.80 |
|  |  |  | 63.5 |  | 1.78 | 3.53 |
